# No impact of sex or testosterone treatment on wheel running in a rat model of inflammatory bowel disease

**DOI:** 10.1186/s13293-026-00882-0

**Published:** 2026-04-02

**Authors:** Rebecca M. Craft, Christyne M. Sewell, Christa M. Hickey, Kristen Delevich, Michael M. Morgan

**Affiliations:** 1 Department of Psychology, Washington State University, PO Box 644820, Pullman and Vancouver, WA 99164-4820 USA; 2 Integrative Physiology and Neuroscience, Washington State University, Pullman and Vancouver, WA USA

**Keywords:** Sex differences, Testosterone, Estradiol, Colitis, Gender-affirming hormone treatment

## Abstract

**Background:**

Sex differences in inflammatory bowel disease (IBD) have been reported in humans, and gonadal steroid hormones are implicated in these sex differences. Although pain is a primary complaint among IBD patients, and pain often does not correlate with colon pathology, most animal studies focus on physiological rather than behavioral measures of IBD severity. Thus, the present study determined whether the impact of colitis on global measures of well-being is greater in female than male rats, and whether testosterone ameliorates this impact in females.

**Methods:**

Blank capsules were implanted into gonadally intact adult females and males, and another group of females was implanted with testosterone-filled capsules. Three weeks later, trinitrobenzene sulphonic acid (TNBS) was administered intracolonically to induce colitis. Body weight and continuous, home cage wheel running were measured daily, before and for 10 days after colitis induction. Estrous cycle was monitored for 21 days in a subset of females. At the end of the study, serum testosterone and estradiol were determined, in addition to clitoral/preputial gland size.

**Results:**

TNBS suppressed body weight and wheel running, which partially recovered within 10 days, with no group differences in magnitude or time course of these effects. Serum testosterone was elevated in testosterone-treated compared to control females and did not differ significantly from males, whereas serum estradiol was similar across groups. Testosterone suppressed females’ estrous cycling and increased clitoral gland size. At the end of the study, serum estradiol was found to be correlated with suppression of body weight and wheel running during the previous 10 days.

**Conclusions:**

These results do not support the hypothesis of sex differences in well-being after IBD induction, or that testosterone ameliorates IBD effects in females, but corroborate human and rodent data suggesting that estradiol is associated with IBD severity in both sexes.

## Abstract (plain language summary)

Inflammatory bowel disease (IBD) is more common in women than men. Women IBD patients may also suffer more pain than men IBD patients, and hormone levels may influence pain in both women and men IBD patients. This study tested whether males and females differ in their behavioral response to inflammation of the colon, and whether hormones influence this response. Adult male and female rats were implanted with placebo (blank) capsules, and a separate group of adult females was implanted with continuous-release testosterone capsules, to determine whether testosterone would ameliorate IBD-like effects in females. After 3 weeks, rats were anesthetized and a chemical irritant was infused into the colon to produce inflammation and pain. Body weight and continuous voluntary wheel running were then recorded daily for 10 more days. Rats’ body weight and wheel running dropped for several days and then gradually recovered over the next week. There were no sex differences in these effects, nor did testosterone treatment reduce the negative impact of the colon irritant in females. However, in both sexes, the higher the estradiol level, the more negative the impact was. Contrary to previous rodent studies showing sex differences in some irritant effects on colon *tissue*, this study showed that when *behavior* was measured, females and males responded similarly, and treating females with testosterone did not ameliorate the negative impact of colonic irritation. Further animal studies will be necessary to determine causation – that is, whether *manipulating* estradiol levels can reduce the negative impact of colonic irritation.

## Highlights

Wheel running behavior and body weight loss did not differ between females and males in a rat model of inflammatory bowel disease

Testosterone exposure at male-typical levels increased females’ body weight, suppressed estrous cycling, and increased clitoral gland size, but did not alter wheel running behavior of gonadally intact females

Estradiol was significantly correlated with TNBS effects in both sexes, with higher estradiol levels associated with greater suppression of behavior and body weight

Using global measures of well-being, this study suggests that there are no sex differences in IBD-related effects, and that male-typical levels of testosterone are not protective against IBD-related effects in females

## Background

Inflammatory bowel disease (IBD) affects approximately 5 million people worldwide, with prevalence, deaths and disability-adjusted life years often reported to be greater in women than men [1, 2]. A majority of IBD patients experience abdominal pain, which contributes substantially to decreased quality of life and is a major clinical challenge [3]. Abdominal pain prevalence is reported to be greater in women than men with IBD [4, 5], although the opposite has also been reported [6]. Both testosterone (T) and estradiol may influence IBD prevalence and severity [7, 8]. For example, multiple studies have found a negative correlation between T levels and IBD susceptibility or severity (e.g. [9, 10]), , including in women [8, 11]. Furthermore, rates of IBD were lower among transgender men currently using gender affirming T therapy compared to those who had never or formerly used it [12]. In contrast, exogenous ovarian hormone use was found to increase the risk of IBD in reproductive-age women [13].

In rodent models of IBD, numerous sex differences in histological, visceromotor, and immunological responses have been documented. However, the direction of the sex difference varies by what is measured, and in some cases by the strain of mouse [14–18]. Both T and estradiol have been implicated in sex differences in IBD-like colon pathology [11, 19, 20]. In contrast, pain-related *behaviors* in awake, freely moving animals are rarely assessed in IBD models, despite the fact that abdominal pain is one of the two most “bothersome” symptoms reported by patients [21]. Furthermore, colonic inflammation often does not correlate with abdominal pain in IBD patients: pain also occurs between flare-ups, substantially decreasing patients’ well-being [4]. Thus, modeling pain and well-being is an important aspect of understanding sex differences in and hormonal modulation of IBD. Spontaneous IBD-like pain has been compared in females vs. males in one study: female mice showed more total pain-related behaviors than males after acute intracolonic capsaicin; however, when *persistent* IBD was induced with 7 days of oral dextran sulfate sodium (DSS), males showed more disease progression than females, yet there were *no* sex differences in the total number of spontaneous pain-related behaviors after subsequent intracolonic capsaicin [22]. The roles of T and estradiol in modulating spontaneous pain in preclinical models of IBD have not been examined.

To address this knowledge gap, the present study determined whether: (1) female rats are more likely than males to exhibit persistent IBD-like behavioral depression; (2) male*-*typical levels of T ameliorate pain-related behavioral depression in females; (3) IBD-like severity is correlated with estradiol levels. A key aspect of clinically validated pain evaluation is the extent to which the pain interferes with activities of daily living, such as walking, working, sleeping, etc. (e.g., as in the Brief Pain Inventory used to measure pain in patients, including those with IBD [23]). Home cage wheel running is a low-stress, objective measure obtained in awake rodents that provides a global, clinically relevant measure of well-being [24]. IBD-like behavioral depression was induced with intracolonic trinitrobenzene sulphonic acid (TNBS), which causes inflammation and histological changes consistent with human IBD [25, 26], and decreases rats’ voluntary home cage wheel running for at least 5 days [27]. Given the reported sex differences in, and possible influence of T and estradiol on human IBD, it was hypothesized that the magnitude and duration of TNBS-suppressed wheel running would be greater in female than male rats, that T would attenuate the impact of TNBS in females, and that estradiol levels would be correlated with rats’ response to TNBS. The clinical relevance of T treatment in gonadally intact females was determined by its impact on estrous cycling and clitoral gland size, since T therapy has been reported to reduce menstrual cycling and increase clitoral size in transgender men [28, 29].

## Methods

### Subjects

Sprague-Dawley rats identified as female and male by external genitalia were purchased from Envigo Labs (Livermore, CA), and upon arrival were housed in same-sex pairs under a 12:12 h light: dark cycle for approximately one week prior to beginning the experiment. Thereafter, rats were housed singly, in one of two test rooms (12 cages/test room), with lights off at 1400 or 1430 depending on the test room. Rats were tested in seven cohorts of 12 rats/cohort.

Rats were 47–61 days old when each was singly housed in a home cage with a running wheel. Each rat remained in this cage with *ad libitum* access to food and water except during weighing, vaginal lavage, capsule implantation, saline/TNBS instillation, and animal husbandry. All manipulations except surgery were conducted during the hour before the dark phase began; surgery was conducted during a 3-h period before the dark phase began. All procedures were conducted in accordance with the NIH Guide for Care and Use of Laboratory Animals [30], and were approved by the Washington State University Institutional Animal Care and Use Committee.

### Apparatus

Each rat had continuous home cage access to a running wheel (Tecniplast, Starr Life Sciences Corp, PA). The wheel had a circumference of 1.04 m and was mounted to hang from the top of a standard rat cage. The number of wheel revolutions was recorded continuously except for the last hour of the light phase, when no wheel running data were collected so that animal husbandry and experimental manipulations could be conducted. The number of wheel revolutions was recorded using VitalView software (Starr Life Sciences Corp, Oakmont, PA) on a computer located in a room adjacent to the testing room.

### Hormones and chemicals

Crystalline testosterone (T) was purchased from Steraloids (Newport, RI); T-filled and blank Silastic capsules (0.062 in. i.d./0.125 in. o.d.) were constructed in-house, in 5-mm and 10-mm lengths as previously described [31]. 2, 4, 6-Trinitrobenzene sulphonic acid (TNBS, Millipore Sigma, St. Louis, MO, USA) 30 mg/ml was prepared in a 1:4:5 ratio of TNBS: saline: ethanol. Sterile physiological saline served as the vehicle control.

### Surgeries

To model T exposure as used in gender-affirming T therapy – in which the goal is to achieve and maintain cisgender male levels of T [32], typically in people who retain their ovaries – constant-release T-filled or blank capsules were implanted in gonadally intact female rats at doses that maintain reproductive behaviors and organ weights in orchidectomized male rats similarly to those in gonadally intact male rats [31, 33]. Rats (57–71 days old) were anesthetized with an i.p. injection of ketamine (90 mg/kg) and xylazine (10 mg/kg) (MWI Animal Health, Visalia, CA). Meloxicam (1 mg/kg; Patterson Veterinary Supply) was administered s.c. as a pre-operative analgesic. Constant-release, Silastic capsules were implanted s.c. between the shoulder blades based on body weight (approximately one 10-mm capsule/100 g body weight): Rats weighing 160–210 g were implanted with two 10-mm capsules; rats weighing 211–235 g were implanted with two 10-mm and one 5-mm capsules. Half of the female rats were implanted with capsules containing T and the other half received blank capsules (female control group). Each male was implanted with two blank capsules.

### Behavioral procedure

**Fig. 1:**
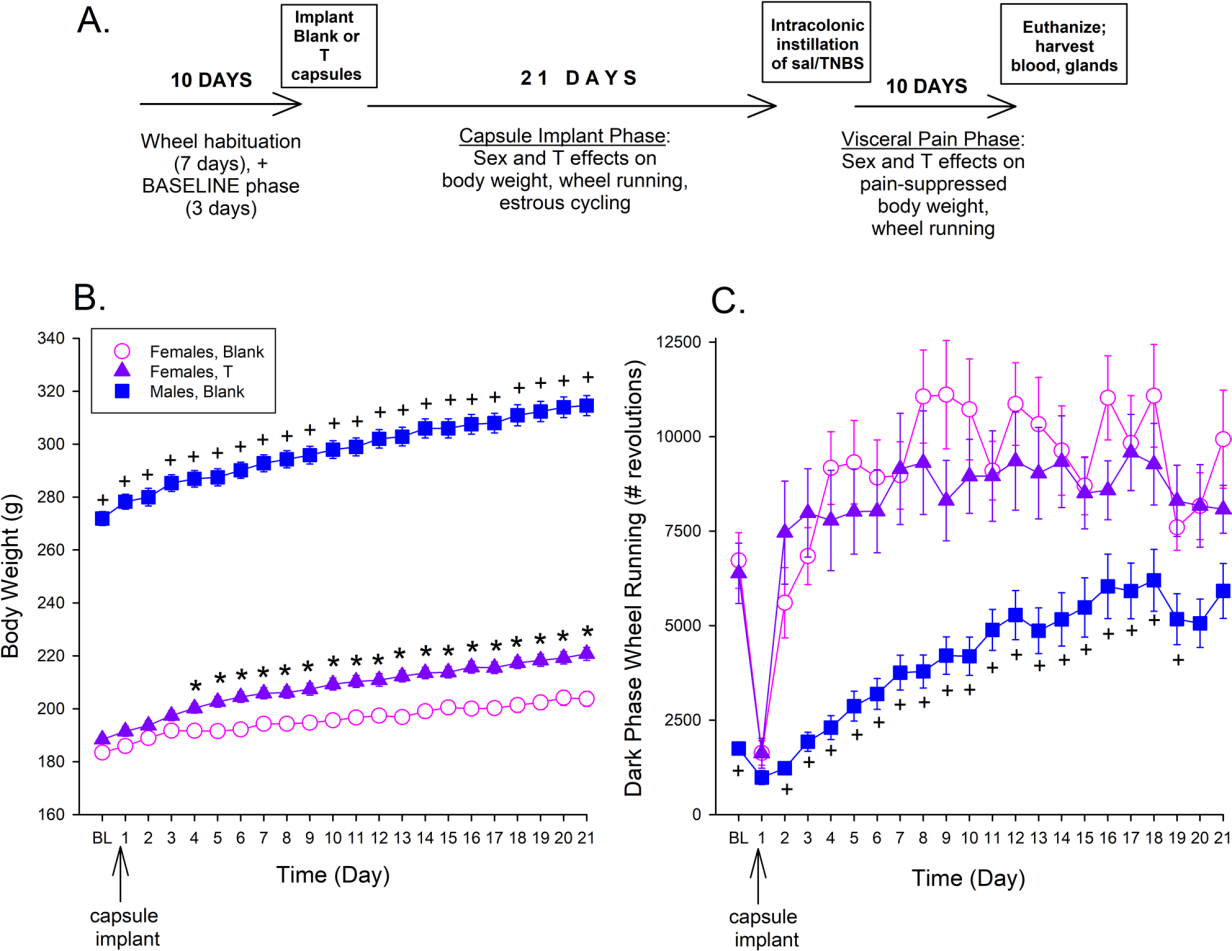
**A**. Experimental Timeline. **B.-C**. Capsule Implant Phase: Impact of sex and testosterone treatment on (**B**) body weight, and (**C**) dark phase wheel running. On Day 1 female rats were implanted with capsules containing no hormone (Blank) or testosterone (T), and male rats were implanted with blank capsules. Each point is the mean ± 1 S.E.M. of 24 Blank- or 25 T-implanted females, or 31 Blank-implanted males. *T-implanted females greater than Blank-implanted females at same time point; ^+^Blank-implanted males different from Blank-implanted females at same time point, *p* ≤ 0.0023, Bonferroni-corrected t test

The experimental timeline is illustrated in Fig. 1A. Rats were acclimated to single-housing with a running wheel for 10 days. Wheel running gradually increases during this habituation period, with a majority of wheel running occurring during the dark phase [34]. Baseline running was defined as the average number of wheel revolutions during the dark phase over the last 3 days of the 10-day habituation period. A matching protocol was then used to assign female rats to each of the 4 female treatment groups (Blank + Saline, Blank + TNBS, T + Saline, T + TNBS), and to assign males to each of the 2 male groups (Blank + Saline, Blank + TNBS), so that mean baseline wheel running was comparable across treatment groups within each sex, at the start of the experiment. Rats were weighed daily in g throughout the experiment.

Near the end of the 3-day baseline period (within the last few hours of the light phase on the third baseline day), rats were anesthetized and blank or T-filled capsules were implanted s.c. (see Surgeries). Rats were returned to their home cages, and wheel running was recorded 23 h/day for the next 3 weeks (“Capsule Implant Phase”).

After the last day of the Capsule Implant Phase, rats were anesthetized with isoflurane during the last hour of the light phase for instillation of 0.6 ml sterile saline or TNBS, administered via PE60 tubing inserted 7 cm into the distal colon [27]. Rats were returned to their home cages, and wheel running was recorded 23 h/day for the next 10 days (“Visceral Pain Phase”). Any rat that lost more than 20% body weight during this phase was euthanized.

Following the Visceral Pain Phase, within the first two hours of the dark phase, rats were euthanized with isoflurane and trunk blood was collected and centrifuged for 20 min at 3200 rpm. Serum was collected from all rats that completed the 41-day study, and stored at -80˚C for later determination of hormone levels. Capsules were removed to confirm number and type (blank or T), and the clitoral glands (females) or preputial glands (males) were removed and stored in 10% formalin for a minimum of 2 weeks before trimming and measuring length and width of the right and left glands in mm, by an experimenter blind to treatment group assignment.

### Estrous cycle monitoring

For 8–9 rats in each of the four female groups, vaginal lavage was conducted daily for up to 21 days, starting 1.5-2 weeks after capsule implantation and continuing 4–10 days into the Visceral Pain Phase. Slides were air-dried and later stained with Giemsa (Sigma Aldrich). Estrous stage was determined via microscope by a reader who was blind to treatment group assignment: proestrus was defined as approximately 75% or more of cells in the sample being nucleated epithelial cells; estrus was defined as approximately 75% or more of cells in the sample being cornified epithelial cells; diestrus was defined as an approximately equal distribution of nucleated and cornified epithelial cells plus leukocytes (diestrus day 1, also known as metestrus), or primarily leukocytes (diestrus day 2) [35].

### Hormone analysis

Serum T and estradiol levels were determined in duplicate, using ELISA kits (IB79106: Immuno-Biological Laboratories, Inc., Minneapolis, MN, and 11-ESTHU-E01: American Laboratory Products Company, Salem, NH) according to the manufacturer’s protocol by a technician blind to treatment group assignment. Intra-assay precision was calculated as the coefficient of variation (CV%) between duplicate wells within each plate. Across standards (20–3200 pg/mL for estradiol), intra-assay CVs ranged from 0.7 to 12%. Inter-assay precision was determined using plate means across two plates, yielding CVs of 1.3–4.2% across the quantifiable range. Kit controls fell within manufacturer-specified acceptable ranges on both plates. Inter-assay CV for controls was 6.2% (high control) and 17.6% (low control). The high intra- and inter-assay precision observed across standards and kit controls indicates strong assay reliability, supporting the interpretation that the large proportion of samples below the minimum level of detection reflects true low estradiol concentrations rather than technical variability.

### Data analysis

To quantify estrous cycling, percent time in proestrus and estrus was estimated as: # proestrus and estrus samples / number of sample days x 100. Percent time in proestrus and estrus was then compared among the female groups (blank vs. T) by ANOVA, with TNBS (saline vs. TNBS) entered as a co-variate, because TNBS might be expected to disrupt cycling but was not a primary variable of interest for this analysis.

Clitoral and preputial gland sizes were estimated by calculating the mean length and width of the right and left glands for each rat, and then calculating the area (mean length x mean width, in mm^2^). Because glands were expected to scale by size of the rat to some degree, gland area was also adjusted by final body weight, for each rat. Unadjusted and body weight-adjusted gland area were each compared among treatment groups (female, blank; female, T; male blank) by ANOVA, with TNBS entered as a co-variate. Glands were harvested from all rats except one female in the T + TNBS group.

Since wheel running occurs primarily during the dark phase, only the number of wheel revolutions during daily 12-h dark phase periods was analyzed. The initial baseline was the mean number of dark phase wheel revolutions over the last 3 days of the 10-day habituation period, calculated for each rat. One to four days before the end of the study, several rats were euthanized due to excessive weight loss after TNBS instillation (3 Females, Blank; 2 Females, T; 1 Male); in these cases, missing body weight and wheel running values were replaced with those obtained on the last day each rat was alive. Body weight and wheel running data were analyzed in two phases. Data during the 3-week Capsule Implant Phase were analyzed by ANOVA, with factors of Treatment Group (Females, Blank; Females, T; Males, Blank) and Day (repeated measure; baseline + 21 days post-capsule implant). Significant treatment group differences were followed by planned comparisons to test for sex differences (females implanted with blank capsules vs. males implanted with blank capsules), and to test for a T effect between the two female groups. Because there were group differences in body weight and wheel running at the end of the Capsule Implant Phase, body weight and wheel running data during the subsequent 10-day Visceral Pain Phase were converted to percent of baseline, for each rat; the second baseline was the mean body weight of the last 2 days, or mean number of dark phase wheel revolutions during the last 3 days of the Capsule Implant Phase. Percent baseline body weight and percent baseline wheel running were each analyzed by ANOVA with factors of Treatment Group, TNBS, and Day (repeated measure).

The mean of the two duplicates was calculated for each serum hormone sample. Hormone levels then were compared among the three treatment groups via 2-way ANOVA, with factors of Treatment Group and TNBS. To determine if estradiol levels at the end of the study were associated with TNBS-suppressed body weight and wheel running during the 10-day visceral pain phase, mean body weight and mean dark phase wheel running during the 10 days after TNBS instillation were calculated for each rat (using percent of baseline values), and the associations between these values and estradiol levels were tested using Pearson correlation analyses.

In the case of serum estradiol, at least 1 of the 2 duplicates from 27 (of 73 total) samples yielded estradiol levels below the minimum level of detection (10 pg/ml). The proportion of samples with at least one duplicate below the minimum level of detection did not differ among treatment groups, but data were also analyzed after imputing mean values that were below the level of detection, using the minimum detectable concentration of 10 pg/ml.

To determine whether the frequency of TNBS-related morbidity, and the frequency of estradiol values below the level of detection differed among the three treatment groups, non-parametric Pearson Chi Square tests were used, because these variables were nominal and neither continuous nor normally distributed.

SPSS version 29 was used for analyses. Post-hoc comparisons were conducted using Tukey’s test, or a Bonferroni-corrected *t* test to compare groups on 22 days. For all repeated measures ANOVAs, Mauchley’s test of sphericity was used to test for homogeneity of variance; if this assumption was violated *and* Greenhouse-Geisser-adjusted p values were > 0.05 (i.e., if the unadjusted p value was *p* ≤ 0.05 but the adjusted p value was > 0.05), then adjusted df, F, and p values are reported. Partial eta squared (η_p_^2^) values are provided as effect size estimates; η_p_^2^ values of 0.01, 0.06, and 0.14 are considered small, medium, and large, respectively [36].

## Results

### Capsule implant phase: sex differences and impact of T in females

Figure 1B shows group differences in body weight during the 3-week Capsule Implant Phase (Treatment Group x Day: F_42,1617_=12.90, *p* < 0.001, ŋ_p_^2^=0.251). Among rats implanted with blank capsules, males gained more weight than females (Sex x Day: F_21,1113_=21.64, *p* < 0.001, ŋ_p_^2^=0.290), and T-implanted females gained more weight than females implanted with blank capsules (T x Day: F_21,987_=10.50, *p* < 0.001, ŋ_p_^2^=0.183). Figure 1C shows that during the Capsule Implant Phase, there were also group differences in wheel running on most days (Treatment Group x Day: F_42,1617_=3.22, *p* < 0.001, ŋ_p_^2^=0.077). Specifically, Blank-implanted males ran less than Blank-implanted females (Sex: F_1,53_=36.42, *p* < 0.001, ŋ_p_^2^=0.407; Sex x Day: F_21,1113_=3.89, *p* < 0.001, ŋ_p_^2^=0.068). However, wheel running did not differ between Blank- and T-implanted females (no T effect or T x Day interaction).

### Visceral pain phase: sex differences and impact of T in females

**Fig. 2:**
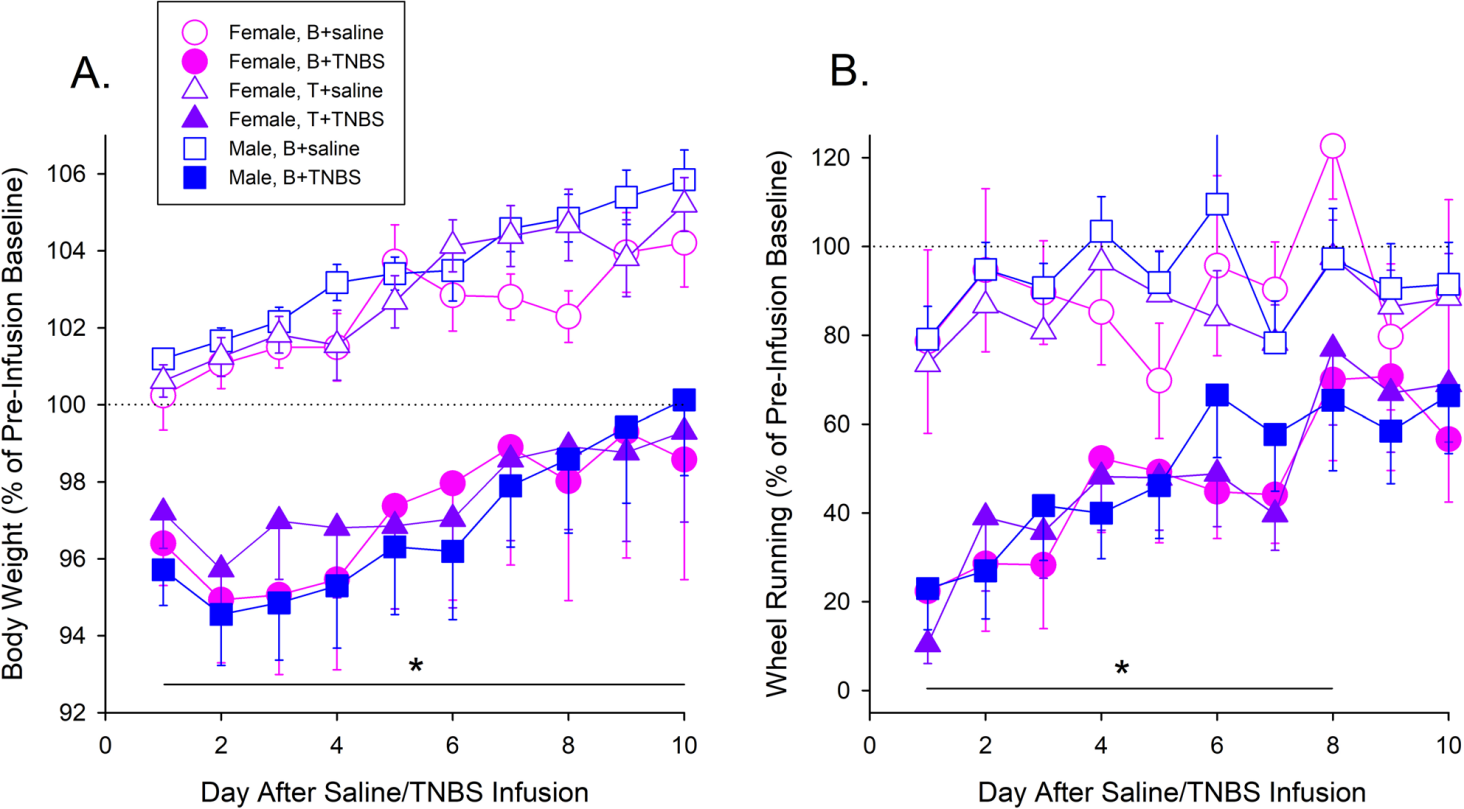
Visceral Pain Phase: Impact of sex and testosterone treatment on (**A**) body weight, and (**B**) dark phase wheel running after intracolonic instillation of saline (open symbols) or TNBS (closed symbols). After the 3-week Capsule Implant Phase, saline or TNBS was instilled into the distal colon. Each point is the mean ± 1 S.E.M. of 12 Blank (**B**)- or 12–13 T-implanted female rats/group, or 15–16 male rats/group. Data were transformed to percent of pre-saline/-TNBS baseline before plotting, because body weight and wheel running differed significantly among groups by Day 21 of the Capsule Implant Phase (see Fig. 1C). TNBS suppressed body weight and wheel running with no significant group differences. *TNBS-treated rats different from saline-treated rats, *p* ≤ 0.005, Bonferroni-corrected t test

Figure 2 shows that relative to saline infusion, intracolonic TNBS caused body weight loss (A) and suppressed wheel running (B) in all groups of rats. On average, rats’ body weight decreased to approximately 95% of baseline within 2 days after TNBS instillation, and recovered partially over the next week (TNBS: F_1,74_=24.64, *p* < 0.001, ŋ_p_^2^=0.250), with no group differences (Fig. 2A). Similarly, TNBS suppressed wheel running to approximately 10–25% of baseline on the first night, and running recovered similarly in all treatment groups over the 10-day period (TNBS x Day: F_9,666_=3.02, *p* = 0.001, ŋ_p_^2^=0.039) (Fig. 2B). Some TNBS-treated rats were euthanized due to excessive weight loss before the end of the study, including 3 of 12 Blank-treated females (25%), 2 of 13 T-treated females (15.4%), and 1 of 16 males (6.3% of sample), but morbidity did not differ statistically among the 3 groups (*Χ*^*2*^ (2, *n* = 41) = 1.94, *p* = 0.38).

### Physiological impact of T treatment in females

**Fig. 3:**
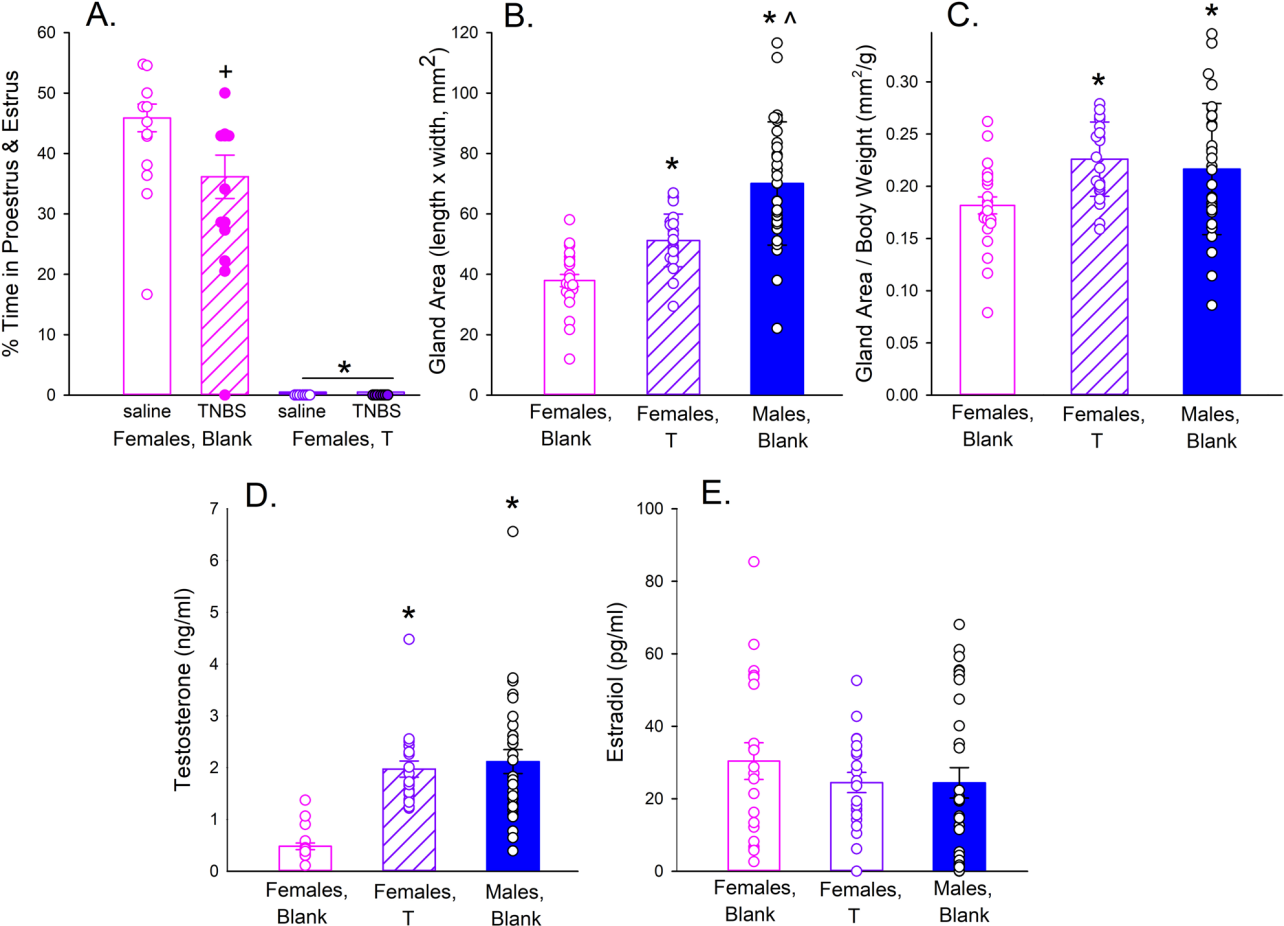
Impact of testosterone treatment on estrous cycling, clitoral/preputial gland size, and serum hormone levels. Female rats were implanted with capsules containing no hormone (Blank) or testosterone (T), and male rats were implanted with blank capsules. Glands and blood samples were obtained at the end of the study, which was approximately 4.5 weeks after capsule implantation and 1.5 weeks after intra-colonic saline or TNBS instillation. **A**. Each bar is the mean + 1 S.E.M. percent time (days) in proestrus or estrus, for Blank- and T-implanted females that were treated with intracolonic saline or TNBS (*n* = 8–9/group). *Significant suppression of estrous cycling in T-implanted females compared to females implanted with blank capsules; ^+^Suppression of cycling in TNBS- compared to saline-treated females implanted with blank capsules, *p* < 0.05, Tukey’s test. **B**. Each bar is the mean + 1 S.E.M. area of the clitoral glands (females: *n* = 24 Blank; *n* = 24 T) or preputial glands (males: *n* = 31). **C**. Same as **B**, but with gland size adjusted by body weight. **D.-E**. Serum testosterone (**D**) and estradiol (**E**). Each bar is the mean + 1 S.E.M. of 21 Blank or 22 testosterone (T)-implanted female rats, or 30 male rats. In all panels, each circle is an individual rat. **B.-E**.: *Significantly different from females implanted with blank capsules; ^Significantly greater than T-implanted females, *p* < 0.05, Tukey’s test

Figure 3A shows that continuous T exposure suppressed estrous cycling compared to blank-implanted females (F_1,31_=341.36, *p* < 0.001, ŋ_p_^2^=0.917). Within the blank-implanted female group, TNBS-treated rats showed fewer days in proestrus and estrus than saline-treated rats (Fig. 3A). For example, on Day 2 of the visceral pain phase, 2 of 12 (16.7%) TNBS-treated control females were in proestrus or estrus, compared to 4 of 12 (33.3%) saline-treated control females.

Figure 3B shows the size of the clitoral glands (females) and preputial glands (males) at the end of the study; because gland area is expected to increase with body size, gland area was also adjusted by body weight (Fig. 3C). Unadjusted gland area differed significantly among the three treatment groups (F_2,75_=33.25, *p* < 0.001; ŋ_p_^2^=0.470). Specifically, gland area was larger in T-implanted females and in males compared to females implanted with blank capsules (Fig. 3B). When gland area was adjusted by body weight, the impact of T remained (Treatment Group: F_2,75_=5.41, *p* = 0.006; ŋ_p_^2^=0.126), and there was no longer any difference between T-implanted females and males (Fig. 3C). TNBS did not affect gland size (F_1,75_=1.08, n.s.; data not shown).

### Serum hormone levels

Figure 3D shows that at the end of the study – approximately 4.5 weeks after capsule implantation – serum T levels were approximately four times higher in T- than in blank-implanted females, and were not different between T-implanted females and males (Treatment Group: F_2,67_=21.50, *p* < 0.001, ŋ_p_^2^=0.391). T levels did not differ significantly between saline- vs. TNBS-treated rats (F_1,67_=0.23, n.s.; data not shown). Estradiol was below the level of detection in at least one duplicate from 8 of 21 (38%) control females, 7 of 22 (32%) T-implanted females, and 12 of 30 (40%) males; these group differences in frequency were not significant (*Χ*^*2*^ (2, *n* = 73) = 0.38, n.s.). Estradiol levels did not significantly differ among the three treatment groups (Fig. 3E) (Treatment Group: F_2,67_=0.62, n.s., ŋ_p_^2^=0.018; no Treatment Group x TNBS interaction).

**Fig. 4:**
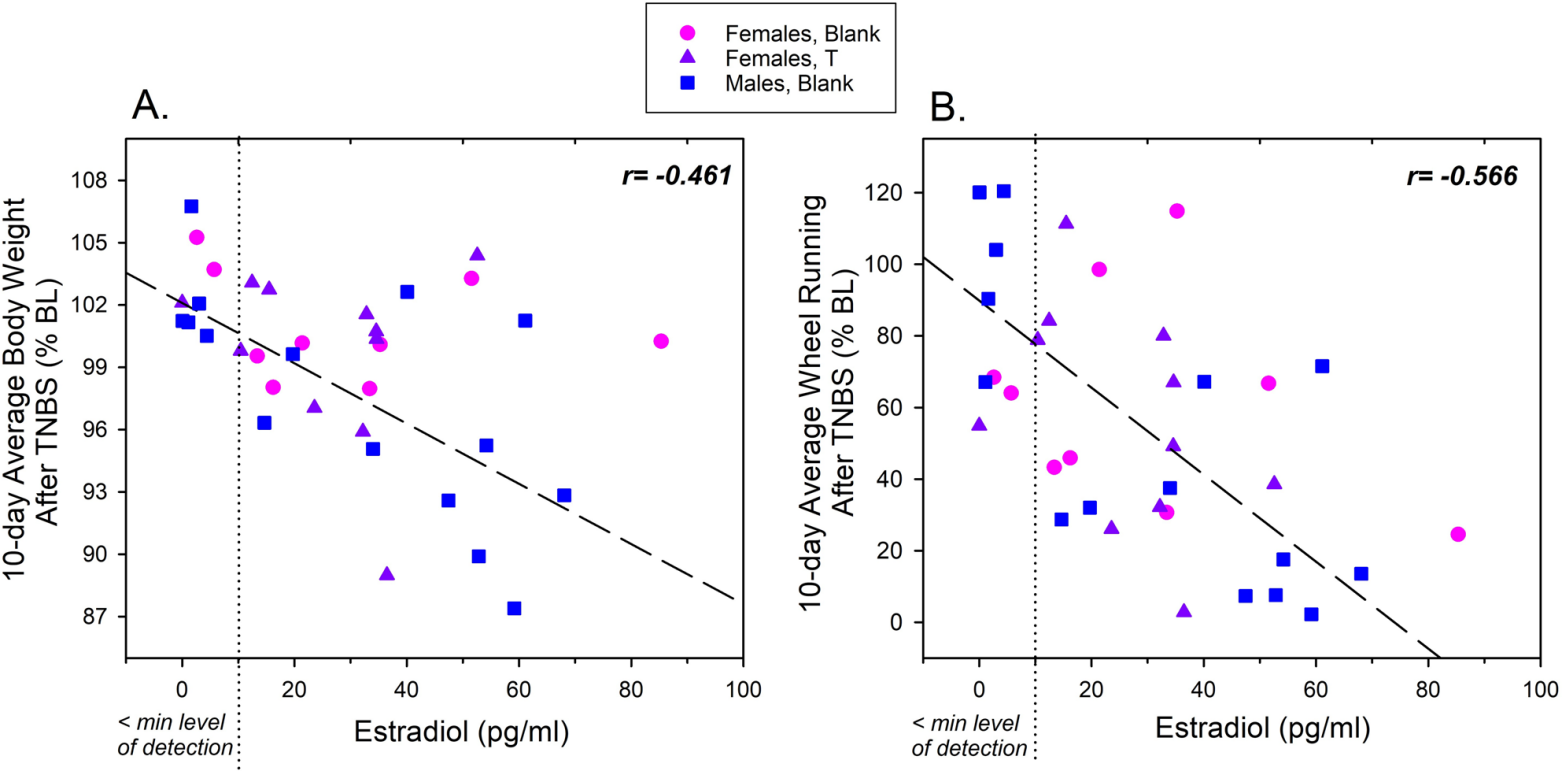
Correlation between serum estradiol at the end of the study with average body weight (**A**), and wheel running (**B**) during the 10-day period after intracolonic instillation of TNBS. Each point represents a single TNBS-treated rat: Blank-implanted females (circles, *n* = 9), T-implanted females (triangles, *n* = 11), or Blank-implanted males (squares, *n* = 15). The minimum level of assay detection for estradiol was 10 pg/ml; values falling below that are to the left of the dotted vertical line. Regression lines are depicted by dashed lines, with Pearson r values are shown in the top right corner of each panel

Correlation analysis showed that estradiol levels at the end of the study were significantly, negatively associated with both average body weight (*r*= -0.461, *p* = 0.005) and wheel running (*r*= -0.566, *p* < 0.001) during the 10 days after TNBS instillation, as shown in Fig. 4A and B, respectively. Statistical results were similar when values below the minimum level of detection were set to 10 pg/ml, for both body weight (*r*= -0.436, *p* = 0.009) and wheel running (*r*= -0.548, *p* < 0.001). Additionally, results were similar when estradiol levels in males alone (the largest treatment group, *n* = 15) were correlated with body weight (*r*= -0.686, *p* = 0.005) and wheel running (*r* = − 0.737, *p* = 0.002). Correlations were negative but not statistically significant when each of the female groups alone were analyzed, perhaps due to smaller sample sizes (control females, *n* = 9; T-implanted females, *n* = 11). There was no correlation between estradiol levels and either body weight or wheel running in saline-treated controls, in any group (data not shown).

## Discussion

The novel findings in this study are: (1) intracolonic TNBS significantly suppressed dark-phase, home cage wheel running and body weight in both females and males, and these global measures of well-being partially recovered over 10 days; (2) although males ran less than females before TNBS infusion, there were no sex differences in TNBS-induced suppression or recovery of wheel running or body weight; (3) T treatment at male-typical levels, which increased body weight and clitoral gland size and suppressed estrous cycling, did not alter females’ responses to TNBS; (4) serum estradiol but not T was significantly, negatively correlated with TNBS effects (i.e., higher estradiol levels were associated with less wheel running and lower body weight after TNBS).

### Sex differences in TNBS-induced behavioral depression and weight loss

We previously showed that intracolonic TNBS suppressed wheel running for approximately one week in adolescent and adult female rats [27]. The present study extends this finding to males, and to females treated with exogenous T at male-typical levels. The magnitude and duration of TNBS-induced suppression of wheel running and body weight did not differ significantly between control (blank-implanted) females and males. Morbidity – excessive weight loss requiring euthanasia – was greatest in the control female group, although this sex difference was not statistically significant. Previous sex comparisons using the TNBS model have focused on the colon rather than on behavior. For example, TNBS produced more severe colitis in male than female B6.129 S mice as measured by the extent of colonic erosion, necrosis, and inflammatory infiltrate [17]. In contrast, TNBS induced similar tissue damage and colon shortening in male and female C57BL/6 mice, but greater plasma extravasation and adrenal weights in females than males [16]. The visceromotor response to colonic distension measured 3 days after TNBS administration was greater in male than female guinea pigs [15]. The other most frequently used model of persistent colon inflammation is oral DSS administration. In the only *behavioral* study assessing sex differences in DSS-induced hypersensitivity (to intracolonic capsaicin), no sex differences were observed in total pain-related behaviors or in referred abdominal hypersensitivity, although males lost a greater percentage of body weight than females (Swiss-Webster mice: [22]). In physiological studies, greater DSS-induced colonic inflammation was found in male than female CD-1 mice [18], and the opposite sex difference was found in C57BL/6 mice [19]. Thus, genotype [16] and specific endpoints measured (histological, electrophysiological, behavioral) may contribute to disparate sex difference results within and across studies. In the present study, TNBS suppressed cycling in females (similar to [37]), perhaps decreasing the likelihood of observing sex differences in wheel running, since female rats in proestrus and estrus exhibit more pain-related behavior and visceromotor response than females in metestrus and diestrus, after colorectal distension [20, 38, 39]. Although the lack of sex differences in IBD-related behavioral suppression in outbred rats in the present study agrees with previously reported results in an outbred strain of mouse in which several spontaneous pain-related behaviors were totaled [22], the latter study did report sex differences in some specific pain-related behaviors. Thus, it is likely that multiple variables influence whether sex differences are observed, perhaps especially what outcome measures are included.

### Effects of continuous T exposure on TNBS-induced behavioral depression and weight loss

In the present study, exogenous T treatment of gonadally intact females did not reduce maximal decreases in or promote recovery from TNBS-induced behavioral depression and weight loss. T-implanted and control females responded similarly to TNBS despite substantial differences in circulating T. Morbidity was slightly but not significantly lower in T-implanted compared to control females. There are no previous preclinical IBD studies that examined the impact of male-typical levels of T on IBD-related pain in females. However, transgender men and gender-diverse people assigned female at birth who use gender-affirming T therapy reported less pelvic pain (which included IBD-related pain) than those not using or formerly using T [12]. Additionally, endogenous T level was found to be protective against developing IBD in women [8], as well as inversely correlated with pelvic pain in women with dysmenorrhea [40]. Given that human studies are necessarily correlational or rely on self-report that is often retrospective, further animal studies will be useful to determine the impact of exogenous T on IBD pain-related behaviors in females. Additionally, longitudinal studies in larger samples of transgender men are needed to clarify under what circumstances T, at female- to male-typical levels, influences IBD-related pain and quality of life.

### Estradiol association with TNBS-induced behavioral depression and weight loss

The present study also showed a significant correlation between estradiol levels and TNBS-induced suppression of body weight and wheel running. That is, higher serum estradiol was associated with lower body weight and less wheel running during the 10 days following TNBS administration. This correlational finding agrees with some but not all previous IBD studies in which estradiol was *manipulated*. For example, DSS-induced colitis – as assessed by body weight loss and colon pathology – was more severe in gonadally intact female mice compared to ovariectomized females, and estradiol replacement worsened colitis in ovariectomized females [41]. Pregnancy-like doses of estradiol given to intact female mice also worsened DSS-induced colitis, although the opposite was found using a different colitis model [42]. Another study also reported that estradiol replacement reduced DSS-induced colitis in gonadectomized mice of both sexes [19]. However, pain-related *behaviors* after TNBS or DSS were not measured in any estradiol manipulation studies we could find. The role of estradiol in IBD severity in humans is also unclear, with many studies relying on self-report (recall) of IBD symptom severity during previous periods of hormone fluctuation (e.g. [43]), , or comparing the incidence of IBD flare-ups between exogenous hormone users and non-users, with some studies concluding that exogenous hormone use is protective [44], and others that it increases risk [45].

Recent studies suggest that some discrepancies regarding estradiol and IBD may be related to the opposite impact of colonic estrogen receptor types (ERs). ERɑ is upregulated in both male and female IBD patients [46]; whereas estradiol acting at ERɑ is believed to worsen DSS-induced colitis, ERß activation can ameliorate it [47, 48]. Thus, if the relative number of ER types changes over time after IBD onset, the impact of estradiol on IBD symptoms would be expected to change. Future animal studies will be useful for distinguishing among the roles of multiple ERs in IBD-related pain, and testing at multiple time points after IBD induction will be crucial. Importantly, the current estradiol results are correlational only, and so future studies in which estradiol is explicitly manipulated in gonadally intact females and males are crucial.

### Continuous T exposure in female rats as a physiological model of gender affirming T therapy

Serum T levels in female rats implanted with T capsules were very similar to those in control males, which models the goal in transgender men of achieving and maintaining blood levels of T in the cisgender male range [32]. T treatment decreased serum estradiol slightly but not significantly, similar to what has been reported in transgender men using gender-affirming T therapy who do not undergo oophorectomy [49], and in a rat model of T therapy [50]. It should be noted that estradiol levels in control females were somewhat low in the present study, which likely reflects the fact that estrous cycling was a bit suppressed; estradiol levels in metestrous and diestrous females are typically ≤ 50% of those in proestrous females [51–53].

In the present study, T eliminated estrous cycling. This finding aligns with previous rodent studies [54, 55], and with the amenorrhea reported by transgender men initiating T treatment: more than half ceased menstruating within 3–6 months [28, 56, 57]. Suppression of estrous (and menstrual) cycling by exogenous T is due to negative feedback on the hypothalamus-pituitary-gonadal axis, which suppresses cyclic release of follicle- and luteinizing-stimulating hormones [58].

Clitoral enlargement is another commonly reported effect of gender-affirming T therapy [59]. In rodents, weeks-long T treatment has been shown to increase external “clitoral structure” in adult female mice [55] and to non-significantly increase clitoral diameter in adult female rats [50]. In the present study, clitoral glands were measured post-mortem, because measurement of these considerably larger structures associated with the clitoris allows for a more accurate assessment of T effect than does measuring the clitoris in live rats. Exogenous T significantly increased clitoral gland size in female rats, and when adjusted for body size, clitoral gland size in T-implanted females was comparable to preputial gland size in males. Together with reduced uterine weight and estrous cycling reported in T-treated female rodents [50, 54, 55]^; present study^, clitoral gland enlargement provides clear evidence of physiological masculinization in response to exogenous T.

### Limitations and significance

In the present study, female and male rats responded similarly to TNBS-induced colitis. The lack of sex differences in colitis severity when using global measures of well-being agrees with a previous preclinical behavioral study, but disagrees with some preclinical studies that focused solely on physiological measures of colitis, as well as some human studies in which greater pain prevalence is reported by women compared to men IBD patients. Given that abdominal pain is a primary complaint among IBD patients, more comprehensive characterization of pain is called for in future preclinical studies. Using continuous wheel running is advantageous because it is conducted in the home cage so is less stressful than evoked-pain measures; it is automated so not subject to experimenter bias; and suppressed running reflects spontaneous rather than evoked discomfort – spontaneous pain being historically unaddressed in animal studies [24]. Although continuous home cage wheel running is reliably depressed by many types of pain manipulations in rodents [24], wheel running also can be decreased by other manipulations that may not be explicitly painful, such as stress, fatigue, and immune activation [60–62]. In fact, single-housing, which is technically necessary to accurately measure individual continuous wheel running behavior, may produce a stress response that complicates interpretation of the TNBS response. For example, in male rats, single-housing slowed recovery of wheel running after hindpaw inflammation, compared to pair-housed controls [63]. Finally, a history of exercise may ameliorate colitis [64], introducing another factor to consider when interpreting the current results. For example, the fact that females run more than males may lessen TNBS impact in females compared to males, perhaps compensating for greater pain that might be observed in sedentary females than males. Assessment of behaviors that more specifically reflect IBD-like pain in single- vs. group-housed, exercising vs. sedentary females and males is needed.

The correlation between estradiol and IBD severity in both sexes agrees with some animal and human data indicating that estradiol worsens IBD, but this must be confirmed in future studies in which estradiol and its receptor-specific actions are explicitly manipulated, particularly in males. Finally, the finding that exogenous T at male-typical levels does not alleviate colitis severity in gonadally intact females suggests that gender affirming T therapy is not likely to protect against IBD. Given that this is the first study to model the impact of T therapy at male-typical levels on TNBS-suppressed behavior in female rats, more research will be required to test the reliability of this novel finding, including determining whether T treatment *after* colitis induction may alleviate pain (i.e., modeling transgender men who have IBD before they initiate hormonal transition). The model of T therapy used herein recapitulates a number of physiological effects that mirror those seen in transgender men using gender affirming hormone therapy. Combined with several other recent models of gender affirming hormone therapy, the current findings provide a preliminary step towards advancing transgender medicine [65], including in the area of pain [66].

## Conclusions

Overall, the current results do not support the hypotheses that females are more susceptible than males to suppression of body weight or daily activity after colitis induction, or that T at male-typical levels ameliorates these effects in females. However, the correlational data agree with previous research that implicates estradiol as a modulator of IBD severity in both sexes.

## Data Availability

The datasets analyzed during the current study are available from the corresponding author on reasonable request.
